# Erasing the Homunculus as an Ongoing Mission: A Reply to the Commentaries

**DOI:** 10.5334/joc.117

**Published:** 2020-09-10

**Authors:** James R. Schmidt, Baptist Liefooghe, Jan De Houwer

**Affiliations:** 1 LEAD-CNRS UMR 5022, Université Bourgogne Franche-Comté (UBFC), FR; 2 Department of Psychology, Utrecht University, NL; 3 Department of Experimental Clinical and Health Psychology, Ghent University, BE

**Keywords:** computational modelling, neural networks, episodic memory, binding, switch costs, feature integration, task-rule congruency, instruction implementation, goals

## Abstract

In our recent article (Schmidt, Liefooghe, & De Houwer, 2020, this volume), we presented an adaptation of the Parallel Episodic Processing (PEP) model for simulating instruction following and task-switching behaviour. In this paper, we respond to five commentaries on our article: Monsell & McLaren (2020), Koch & Lavric (2020), Meiran (2020), Longman (2020), and Pfeuffer (2020). The commentaries discuss potential future modelling goals, deeper reflections on cognitive control, and some potential challenges for our theoretical perspective and associated model. We focus primarily on the latter. In particular, we clarify that we (a) acknowledge the role of cognitive control in task switching, and (b) are arguing that certain task-switching effects do not serve as a good measure of said cognitive control. We also discuss some ambiguities in terminological uses (e.g., the meaning of “task-set reconfiguration”), along with some future experimental and modelling research directions.

## Smash the Control Machine?

Koch and Lavric (2020) suggest that we “are on a sort of ‘mission’ against cognitive control of task-set[s]”. This is untrue. As Longman (2020) reiterates well, one of our aims was to implement some executive processes in a less ambiguous (or “homuncular”) fashion (e.g., instead of referring to vague concepts like “task parameters” or “control settings”). This is what we refer to when we talk about “erasing the homunculus”, which is a quest shared by many researchers (Frings et al., 2020; Hommel & Colzato, 2015; Logan, 1990; Monsell & Driver, 2000; Verbruggen, McLaren, & Chambers, 2014). Within this ongoing mission of erasing the homunculus, we extended the Parallel Episodic Processing (PEP) model (Schmidt et al., 2020) by assuming that participants retrieve goals from exemplar memory on the basis of instructed cue-goal pairings (cf., Schneider & Logan, 2005).

Hypothesized influences of cognitive control (i.e., processes relying on the executive system) on the size of the switch cost also does not argue against our perspective. For example, children with attention deficit produce a reduced switch cost, which is in turn reduced to normal with methylphenidate treatment (e.g., Kramer, Cepeda, & Cepeda, 2001). Meiran (2020) notes that impaired attentional control (a process relying on the executive) may lead to a larger switch cost because attention deficit children rely more on episodic memory, exaggerating the binding biases. This explanation is consistent with our message: cognitive control is important, but the switch cost itself is primarily due to binding biases. In this sense, we are sympathetic with the view of Meiran that automatic effects are often a “side effect” of control (see Meiran, Liefooghe, & De Houwer, 2017).

Similarly, the observation that switch costs are absent when participants are given compound cue instructions (i.e., rather than abstract task instructions; Dreisbach, Goschke, & Haider, 2006, 2007; Forrest, Monsell, & McLaren, 2014), can also be accommodated by PEP by assuming that executive processes impact on the operation of memory and binding processes. In this case, the goal of the participant is different and within PEP this could be implemented by simply coding the cue-target-response combinations into the instructions, rather than cue-goal and decision-response combinations. The PEP model currently does not implement compound-cue target instructions, but potentially could (also potentially relevant for some findings in the contingency learning literature; e.g., Mordkoff & Halterman, 2008).

Longman (2020) discusses a series of eye tracking experiments in which evidence of *attentional inertia* is observed (e.g., Longman, Lavric, Munteanu, & Monsell, 2014; Longman, Lavric, & Monsell, 2016, 2017). The cues indicated to participants not only which task to perform, but also which spatial location the target is in. Participants showed not only a standard switch cost, but also a bias to reattend the last target location on task switches. Though we did not consider spatial attention and these eye-tracking studies did not fully control for binding biases, this attentional-inertia effect is not inconsistent with the goal-inertia of the PEP model. That such attentional-inertia effects can be eliminated by using explicit location cues can also be implemented in our model, as simple retrieval of “where to look” on the basis of the cue likely can be completed much faster than “what task to do and in what location is the target for said task”. What is clear, of course, is that the PEP model has not (yet) specified the implementation of task goals other than “do Task X”, which is an interesting direction for future modelling research.

## Direct Causes

We assume (and model) executive influences on behaviour, but our primary goal was to explore to what extent certain key phenomena (effects) are a *direct* result of and, more importantly, could provide an index of such control. Whether cognitive control is involved in a general way in a paradigm that produces a specific experimental effect and whether this experimental effect is a direct proxy of a cognitive-control operation are two very different questions. With respect to task switching, we are positive that cognitive control is involved when switching tasks. In PEP, this involvement exists, but is (at least primarily) not directly responsible for most of what the model produces. Task-set reconfiguration (TSR) or task-set inertia (TSI) accounts, on the other hand, are driven by the assumption that changing tasks requires the (re)configuration of a task-set (similar to our account), which induces a switch cost (dissimilar to our account). Traditionally, the switch cost is thus considered as the result of TSR, TSI, or both, which are thus specific cognitive operations involved in the change of task-sets.

Of course, it has been long appreciated that other biases exist and that the switch cost is therefore not a process-pure measure of (re)configuration (e.g., Arrington & Logan, 2004; Logan & Bundesen, 2003, 2004; Logan & Schneider, 2006; Monsell & Mizon, 2006; Schneider & Logan, 2005), which is echoed by Koch and Lavric (2020) and Monsell and McLaren (2020). However, part of our endeavour (initially in Schmidt & Liefooghe, 2016; and subsequently in Schmidt et al., 2020) was to assess the *joint contribution* of these biases to the switch cost (i.e., controlling for everything at once). This joint contribution is substantial and, in our view, most of the switch cost is explained by biases not directly related to the change in the task. In addition, we used a best-case design, where the tasks were set up to produce the largest possible switch cost (e.g., short CTIs, arbitrary cues, etc.). However, although we provided the best possible conditions for TSR or TSI to magnify the switch cost (e.g., when no preparation is possible and proactive interference is maximal), the variance left unexplained by generic binding processes was small.

The systematic biases that we and others have observed in the switch cost raise some important questions. Manipulations that increase (or decrease) the size of the switch cost may actually be influencing simpler binding processes rather than the cost of switching tasks. Similar ambiguities have emerged in the attentional control literature (for a consensus paper, see Braem et al., 2019). Generic executive processes may play a role in task switching, but our work presents a new perspective on executive control and highlights other biases that may have more explanatory power in explaining task switching phenomena than previously assumed.

## Ambiguity in Definitions

One point of disagreement with our article seems to be how to describe the functioning of the PEP model conceptually. Traditionally, TSR was defined as a discrete step process that occurs, not necessarily completely, before stimulus processing (e.g., Meiran, 1996; Rogers & Monsell, 1995). TSI was proposed as an alternative (e.g., Allport, Styles, & Hsieh, 1994; Gilbert & Shallice, 2002; Meiran, Kessler, & Adi-Japha, 2008), with the key distinguishing feature that resolution of the task-set is said to occur dynamically, in parallel with stimulus identification. However, Koch and Lavric (2020) and Monsell and McLaren (2020) suggest that TSR might be regarded as an incremental dynamic process, consistent with the PEP implementation. This view has been expressed in some recent discussions of TSR (e.g., Monsell, 2015, 2017). The initial assumptions of TSR are no longer tenable and the initial distinction between TSI and TSR has blurred. Indeed, Monsell and McLaren cite an explicit TSI model, Gilbert and Shallice (2002), as an example implementation of dynamic TSR. In this sense, we agree that more recent proposals about TSR are compatible with the PEP model functioning.

Monsell and McLaren (2020) further suggest that (re)configuration is the result rather than the process of changing task-sets. If the result of priming decision options via episodic retrieval is considered a (re)configuration of the task-set, then the PEP model could indeed be considered to (re)configure itself. But this also makes “reconfiguration” nearly theory neutral, consistent with a plethora of competing perspectives (but perhaps excluding the compound-cue retrieval notion in Schneider & Logan, 2005). It is also somewhat ambiguous what exactly it means to call reconfiguration a result: it is neither a behaviour, nor a mental process. In any case, we do not seem to disagree with Koch and Lavric (2020) or Monsell and McLaren (2020) concerning what the PEP model does, only in what we should label said processes. Yet it is also important to stress that while we do similarly assume passive goal carryover in our model, this goal “inertia” is not the primary driving force of the switch cost or other observed effects in the modelled data (e.g., task-rule congruency effects).

## Future Modelling Efforts

Some of the commentaries discuss future potential modelling goals of the PEP model. For example, Pfeuffer (2020) discusses potential broader extensions to the binding domain, exploring, for instance, stimulus-category and stimulus-response binding effects (e.g., Horner & Henson, 2011; Moutsopoulou, Yang, Desantis, & Waszak, 2015; Pfeuffer, Moutsopoulou, Pfister, Waszak, & Kiesel, 2017), action-effect learning (e.g., Elsner & Hommel, 2001, 2004), and the relation between short-term binding and long-term learning effects (e.g., Giesen, Schmidt, & Rothermund, 2020; Moeller & Frings, 2017a, 2017b; Schmidt, Giesen, & Rothermund, 2020).

Koch and Lavric (2020) and Monsell and McLaren (2020) rightly point out that we did not simulate the reduction in switch cost (RISC) with increasing cue-target intervals (CTIs; e.g., Meiran, Chorev, & Sapir, 2000). RISC has frequently figured into discussions of the relative explanatory power of TSR and TSI (Meiran, 1996; Monsell & Mizon, 2006). Monsell and McLaren further indicate that we did not simulate the residual switch cost (Rogers & Monsell, 1995): a smaller but non-zero switch cost with a long CTI, which suggests that anticipatory TSR or inertia has limits (i.e., is not successful in reconfiguring the task entirely before stimulus onset). We did not report simulations of RISC and residual switch costs, as our work suggests a new ambiguity in what reduces with longer CTIs. For instance, the RISC could be due to a reduction or elimination of binding effects with a longer CTI (e.g., see Frings, 2011; Moeller & Frings, 2017a) and the “true” switch cost might not be reduced at all. Alternatively, it could be that the “true” switch cost is eliminated *entirely*, and the remaining “switch cost” is actually a “residual binding effect”. Thus, it is unclear (for the moment) what an appropriate model fit would look like. With new participant data (e.g., a study like that in Schmidt & Liefooghe, 2016, but with varied CTIs), we may be able to address these questions.

## Conclusions

The PEP model presents a novel perspective on task switching. On the one hand, we do not argue against a role of executive processes on behaviour. We do, however, implement some of these executive processes in a less ambiguous (or “homuncular”) fashion. Our work also highlights that these executive processes may not be directly responsible for many of the behavioural effects observed in task switching. Simpler binding biases, though already well-known, may have much more substantial influences on behaviour than previously assumed. We are encouraged by the interest shown in our work by the commentators and hope that future research will serve to fractionate the homunculus even further and to further explore binding and its relation to task switching.
